# PERSPECTIVES OF OLDER ADULT STAKEHOLDERS ON THE IMPACT OF COMMUNITY-BASED EXERCISE

**DOI:** 10.1093/geroni/igz038.2932

**Published:** 2019-11-08

**Authors:** Lekaa Elhajjmoussa, Allison M Mays, Katrina B Rosales, Sonja L Rosen

**Affiliations:** 1 Cedars Sinai Medical Group, Beverly Hills, California, United States; 2 Cedars-Sinai, Los Angeles, California, United States; 3 Cedars-Sinai Medical Center, Los Angeles, California, United States

## Abstract

This focus-group study aims to identify the perceptions of older adults (>50 years) who participated in community-based exercise classes as part of the Leveraging Exercise to Age in Place (LEAP) Study. LEAP enrollees participate in community-based classes that include Tai Chi, EnhanceFitness, Arthritis Exercise, and the Healthier Living Workshops. Nine LEAP participants attended a focus group at Cedars-Sinai Medical Group. The focus group included quantitative and qualitative questions in both a verbal discussion format and a written questionnaire that examined the effects of liaisons, social connections, and incurred changes as a result of participating in LEAP classes. Participants endorsed liaisons within the healthcare system, including physician referrals and communication with a community health coach, as aiding in their decision to participate in health classes. Participants cited positive changes in their physical states, such as increases mobility and decreases in pain, and positive psychological changes, such as increases in energy and socialization, post LEAP completion. Among the ideas and critiques noted by the focus group attendees was the desire to have LEAP classes become part of communities permanently, and to have longer exercise class session duration. These findings suggest that direct merging of liaisons within the healthcare system and community-based exercise programs for older individuals, such as those offered by LEAP, is an effective way to positively influence older patient outcomes both physiologically and psychologically. These results call for future research that focuses on how healthcare systems and community programs can work together to maximize positive patient outcomes for older individuals.

